# Comparison of Chloroplast Genomes among Species of Unisexual and Bisexual Clades of the Monocot Family Araceae

**DOI:** 10.3390/plants9060737

**Published:** 2020-06-11

**Authors:** Abdullah, Claudia L. Henriquez, Furrukh Mehmood, Iram Shahzadi, Zain Ali, Mohammad Tahir Waheed, Thomas B. Croat, Peter Poczai, Ibrar Ahmed

**Affiliations:** 1 Department of Biochemistry, Faculty of Biological Sciences, Quaid-i-Azam University, Islamabad 45320, Pakistan; abd.ullah@bs.qau.edu.pk (A.); mfarrukh@bs.qau.edu.pk (F.M.); shahzadiiram33@gmail.com (I.S.); xainsolangee@gmail.com (Z.A.); tahirwaheed@qau.edu.pk (M.T.W.); 2 Department of Ecology and Evolutionary Biology, University of California, Los Angeles, CA 90095, USA; chenriquez@ucla.edu; 3 Missouri Botanical Garden, 4344 Shaw Blvd., St. Louis, MO 63110, USA; Thomas.croat@mobot.org; 4 Botany Unit, Finnish Museum of Natural History, University of Helsinki, P.O. Box 7, FI-00014 Helsinki, Finland; 5 Alpha Genomics Private Limited, Islamabad 45710, Pakistan

**Keywords:** Araceae, chloroplast genome, substitutions, gene evolution, inverted repeats, contraction and expansion, phylogenetics

## Abstract

The chloroplast genome provides insight into the evolution of plant species. We de novo assembled and annotated chloroplast genomes of four genera representing three subfamilies of Araceae: *Lasia* *spinosa* (Lasioideae), *Stylochaeton bogneri*, *Zamioculcas zamiifolia* (Zamioculcadoideae), and *Orontium* *aquaticum* (Orontioideae), and performed comparative genomics using these chloroplast genomes. The sizes of the chloroplast genomes ranged from 163,770 bp to 169,982 bp. These genomes comprise 113 unique genes, including 79 protein-coding, 4 rRNA, and 30 tRNA genes. Among these genes, 17–18 genes are duplicated in the inverted repeat (IR) regions, comprising 6–7 protein-coding (including trans-splicing gene *rps*12), 4 rRNA, and 7 tRNA genes. The total number of genes ranged between 130 and 131. The *inf*A gene was found to be a pseudogene in all four genomes reported here. These genomes exhibited high similarities in codon usage, amino acid frequency, RNA editing sites, and microsatellites. The oligonucleotide repeats and junctions JSB (IRb/SSC) and JSA (SSC/IRa) were highly variable among the genomes. The patterns of IR contraction and expansion were shown to be homoplasious, and therefore unsuitable for phylogenetic analyses. Signatures of positive selection were seen in three genes in *S. bogneri,* including *ycf*2, *clp*P, and *rpl*36. This study is a valuable addition to the evolutionary history of chloroplast genome structure in Araceae.

## 1. Introduction

The chloroplast is an important double membrane-bounded organelle that plays a crucial role in photosynthesis and metabolism of fatty acids and amino acids in plant cells [1]. Chloroplasts contain a genome that replicates independently of the nuclear genome [1,2] and mostly exhibits a quadripartite structure in which a pair of inverted repeats (IRa and IRb) separate large single-copy (LSC) and small single-copy (SSC) regions [1,2,3,4]. However, in some plant lineages, exceptions to the typical quadripartite structure are reported. For example, in Taxodiaceae [5] and Fabaceae [6], the IR has been lost; in Pinaceae, the IRs have been excessively reduced [7]; and in Pothoideae (another subfamily of Araceae), the SSC has been greatly reduced [8]. Moreover, a mixture of linear and circular chloroplast genomes have also been described [9]. 

The structure of chloroplast genomes is conserved regarding gene organization, gene content, and intron content [1,10,11,12,13]. However, large-scale events of gene rearrangement, gene loss/generation of pseudogenes, and intron loss are also reported in various plant lineages [11,14,15,16,17,18]. Inverted repeat contraction and expansion in chloroplast genomes create pseudogenes, cause gene duplication, or convert duplicates into single-copy genes [11,12]. Many other types of mutational events also take place within chloroplast genomes, including insertions–deletions (indels), substitutions, tandem repeat variations, and variations in number and type of oligonucleotide repeats [12,19,20,21]. The uniparental inheritance—paternally in some gymnosperms [22] and maternally in most angiosperms [23]—along with adequate levels of polymorphism [1,12,24] make the chloroplast genome suitable for studies of evolution, domestication, and biogeography [1,25]. Specific regions of chloroplast genomes, called mutational hotspots, are predisposed to mutations and show high polymorphism, which make them useful molecular markers for studies of population genetics [24,26]. Recently, complete chloroplast genome sequences were successfully employed to elucidate the phylogenetic relationships among closely related or taxonomically difficult taxa [27,28]. Availability of chloroplast genomes provides quality tools for endangered species conservation, accurate barcoding, and avoiding intentional and unintentional adulteration of medicinal plants [1,29,30]. Moreover, chloroplast genomes play an important role in the identification and determination of the purity of commercial cultivars [1,31]. Chloroplast genome-based studies are also essential in the agricultural sector. The most important step for successful breeding is the selection of closely related and genetically compatible species to introduce the desired traits into cultivars. Recently, chloroplast genome-based studies helped breeders infer the evolutionary relationships between wild relatives and their cultivated crops, thus providing valuable insight into the domestication process [1,24].

Araceae is an ancient and large monocot plant family, comprising 144 genera and 3645 species [32]. Based on flower morphology, the family is roughly divided into two categories: bisexual-flowered species and unisexual-flowered species. The eight subfamilies of Araceae are divided among these categories such that Gymnostachydoideae, Orontioideae, Lemnoideae, Pothoideae, Monsteroideae, and Lasioideae all contain bisexual-flowered species, while Zamioculcadoideae and Aroideae contain unisexual-flowered species (with the exception of *Calla* L.) [33,34,35]. Advancements in high-throughput sequencing (HTS) have made genomic resources available for species of Lemnoideae [36], Monsteroideae [19,37,38], Aroideae [11,39,40], and Orontioideae [41,42]. These studies provide a glimpse into the unique evolutionary events of chloroplast genomes in Araceae, including IR contraction and expansion, gene rearrangement, and signatures of positive selection [11,19,36]. Loss/pseudogenization of important genes has been reported in the genus *Amorphophallus* Blume (Aroideae) [18], and mutations have been tentatively identified as synapomorphies for clades such as the duplication of *rps*15 and *ycf*1 in Lemnoideae [36]. These findings suggest that further sequencing of chloroplast genomes from additional aroid subfamilies, such as Lasioideae and Zamioculcadoideae, and additional species from the subfamily Orontioideae will reveal new insights into the evolution of chloroplast genome structure in Araceae. 

Taxa of the “Unisexual Flowers clade” [33,34] that are of interest include Zamioculcadoideae and the independent genus *Stylochaeton* Lepr. *Stylochaeton bogneri* Mayo is of particular concern since it has been declared an endangered species by the International Union for Conservation of Nature. Hence, the chloroplast genome of this species will be helpful for barcoding and conservation purposes. Zamioculcadoideae and *S. bogneri* are both interesting due to their morphology, which consists of a unique combination of unisexual flowers with a perigone (all other taxa with unisexual flowers lack a perigone) [33].

In the current study, we report de novo assembled and fully annotated chloroplast genomes of four species from three subfamilies of Araceae: *Lasia spinosa* (L.) Thwaites (Lasioideae), *Stylochaeton bogneri* and *Zamioculcas zamiifolia* (Lodd.) Engl. (Zamioculcadoideae), and *Orontium aquaticum* L. (Orontioideae). We performed comparative chloroplast genomics among these species, which will provide better insight into the evolution of chloroplast genomes between unisexual and bisexual clades of Araceae. 

## 2. Materials and Methods 

### 2.1. Sample Collection, DNA Extraction and Sequencing

**Figure 1:**
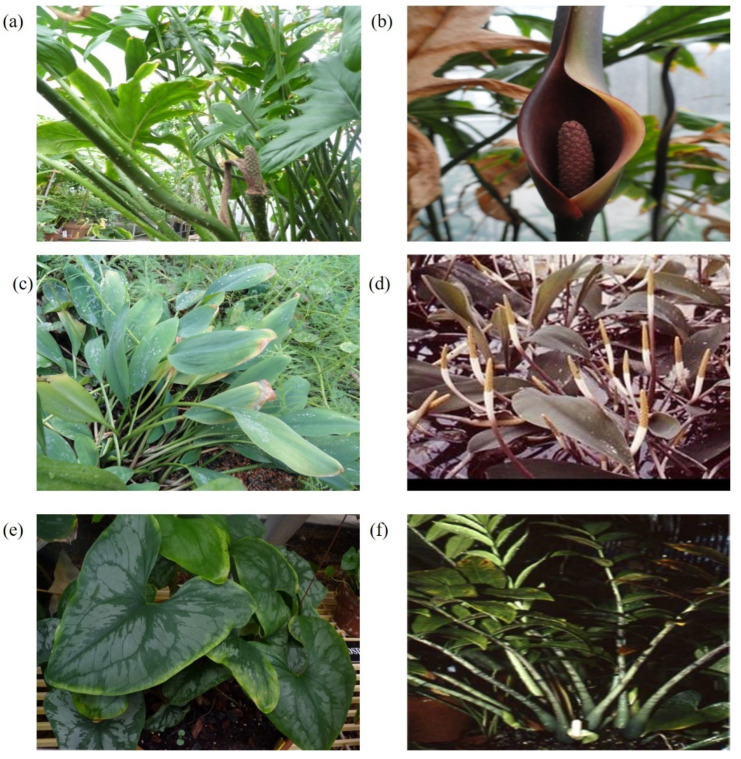
Vegetative and reproductive stages of bisexual and unisexual species. (**a**) *L. spinosa*, tropical Southeast Asian aculeate colony-forming evergreen herbs with deeply pinnatifid hastate-sagittate leaf blades and spadix at antithesis. (**b**) *L. spinosa*, solitary inflorescence of bisexual flowers. (**c**) *O. aquaticum*, temperate east North American seasonally dormant, aquatic herbs with oblong-elliptic blades held above water. (**d**) *O. aquaticum*, numerous bisexual inflorescences held above water level with disintegrated spathe not visible. (**e**) *S. bogneri*, tropical and southeast subtropical African evergreen herb with cordate-sagittate leaf blades. (**f**) *Z. zamiifolia*, tropical east to subtropical southeast African seasonally dormant or evergreen herb with pinnatisect leaf blades and an inflorescence of unisexual flowers with basal female and apical male flowers [43].

We collected fresh and healthy leaves of four species (*L. spinosa*, *S. bogneri*, *Z. zamiifolia*, and *O. aquaticum*) from the Araceae Greenhouse at the Missouri Botanical Garden in St. Louis, Missouri (Figure 1). Whole genomic DNA was extracted from the collected leaves using Qiagen DNeasy Minikit (Qiagen, Germantown, MD, USA), with some modifications following a previous approach [11,19]. DNA quality and quantity were confirmed using 1% agarose gel electrophoresis and Nanodrop (Thermo Fisher Scientific, Waltham, MA, USA). The libraries were constructed following the manufacturer’s protocol for Illumina TruSeq kits (Illumina, Inc., San Diego, CA, USA) in the Pires laboratory at the University of Missouri, Columbia. The Illumina HiSeq 2000 platform was used to sequence qualified libraries from single end with 100 bp short reads at the University of Missouri DNA Core. 

### 2.2. Genome Assembly and Annotation

The sequencing of these genomes generated 3.31 GB (*S. bogneri*) to 11.3 GB (*Z. zamiifolia*) of raw data (Table 1). The quality of the generated short read data was compared among species using FastQC and MultiQC [44,45]. The analyses confirmed high quality of the data, with a high average Phred score ranging from 35.69 to 37.6. The raw data of the four sequenced species were submitted to the Sequence Read Archive of the National Center for Biotechnology (NCBI) under SRA project number PRJNA613281. The generated sequence data were used to de novo assemble chloroplast genomes using Velvet v.1.2.10 [46] by generating contigs with various kmer values of 51, 61, 71, and 81, combined with the de novo assembly option of Geneious R8.1 [47] following previous studies [10,12,48]. The coverage depth analysis was performed by mapping the short reads to their respective de novo assembled chloroplast genomes by BWA (Burrows-Wheeler Aligner) mem [49]. The assembly of the genomes was then validated by visualizing in Tablet [50]. We observed issues at 4–5 points of repetitive regions, therefore, for further validation we used Fast-Plast v.1.2.2 following exactly the same procedure employed for the assembly of other Araceae species [11,19]. This helped us to corroborate the correct sequence at these points. The coverage depth analyses revealed that the average coverage depths of the genomes ranged from 92.7× to 1021×. The de novo assembled chloroplast genomes were annotated by GeSeq [51], whereas tRNA genes were further verified by tRNAscan-SE v.2.0.3 [52] and ARAGORN v.1.2.38 [53] by selecting default parameters. The final annotated genomes were submitted to NCBI under specific accession numbers (Table 1). GB2sequin was used to generate five column tab-delimited files from the annotated genomes for NCBI submission [54]. The circular map of these genomes was drawn using OrganellarGenomeDRAW (OGDRAW) [55].

### 2.3. Characterization, Comparative Analyses and Phylogenetic Inference

We used Geneious R8.1 [47] to compare genomic features and determine amino acid frequency and codon usage. To visualize and compare the junctions of chloroplast genomes, we used IRscope with default parameters [56]. The integrated Mauve alignment [57] of Geneious R8.1 was applied to analyze gene arrangement based on colinear block analyses after removal of IRa from the genomes. 

The Predictive RNA editors for Plant (PREP-CP) [58] program was used to determine RNA editing sites in the chloroplast genomes. We also analyzed microsatellites and oligonucleotide repeats using MISA (MIcroSAtellite) and REPuter, respectively. We determined microsatellites with repeat units as follows: mononucleotide repeats ≥ 10, dinucleotide ≥ 5, trinucleotide ≥ 4, tetranucleotide, pentanucleotide, and hexanucleotide ≥ 3. The forward and reverse oligonucleotide repeats were determined with length ≥ 14 bp with one editing site, initially. Later, we removed all repeats that contained mismatches from the analyses, leaving only those repeat pairs that exhibited 100% similarities, following Abdullah et al. [48].

We determined transition substitutions (*Ts*), transversion substitutions (*Tv*), and their ratio (*Ts/Tv*) in 78 protein-coding genes. For this purpose, we concatenated the protein-coding genes of all four species. The sequences of the concatenated protein-coding genes of *L. spinosa*, *S. bogneri*, and *Z. zamiifolia* were pairwise aligned to *O. aquaticum* by multiple alignment using fast Fourier transform (MAFFT). The substitution types were determined from each alignment in Geneious R8.1 [47]. 

We also determined the rate of synonymous substitutions (*K_s_*), non-synonymous substitutions (*K_a_*), and their ratio (*Ka/Ks*) in 78 protein-coding genes. We extracted and aligned protein-coding genes from all four species. The chloroplast genome of *O. aquaticum* was used as a reference, and the rates of evolution of protein-coding genes were recorded. A similar approach was previously applied in other angiosperms [10,12,17,19,59]. The data were interpreted in terms of purifying selection (*K_a_/K_s_* < 1), neutral evolution (*K_a_/K_s_* = 1), and positive selection (*K_a_/K_s_* > 1). 

A phylogenetic analysis was performed among 30 aroid species and *Acorus americanus* (Acoraceae) as an outgroup. MAFFT [60] on XSEDE v.7.402 in CIPRES [61] was used to align complete chloroplast genomes of 31 of the above species, after removal of one IR. We removed indels events from the alignment to construct a phylogenetic tree based on only substitution mutations, following previous studies [3,11,12,17,19]. The phylogeny was inferred based on this alignment (93,821 nucleotides long, 13,486 parsimony informative sites) using RAxML-HPC BlackBox v.8.2.12 [62] in CIPRES [61]. Table S1 gives details of the species that were used in the phylogenetic analysis. 

## 3. Results

### 3.1. Comparative Genomics among De Novo Assembled Chloroplast Genomes

**Figure 2:**
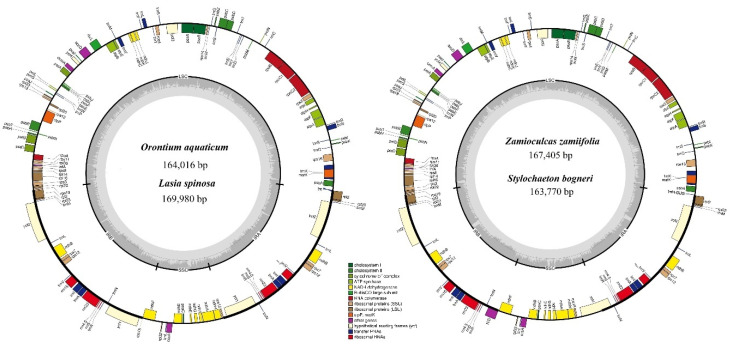
Circular maps of chloroplast genomes. Genes present inside the circle are transcribed counter-clockwise, whereas genes present outside the circle are transcribed clockwise. Genes are color-coded based on functionality. LSC, IRb, SSC, and IRa of the inner circle represent quadripartite structure of genomes.

**Figure 3:**
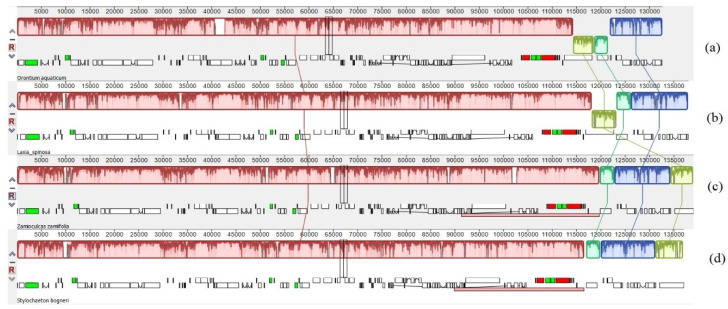
Colinear block-based analyses of gene arrangement in the chloroplast genomes. (**a**) *O. aquaticum*, (**b**) *L. spinosa*, (**c**) *Z. zamiifolia*, and (**d**) *S. bogneri.* The black block: transfer RNA genes, green block: transfer RNA genes with introns, white block: coding genes, and red block: ribosomal RNA genes. Light green and dark green blocks show differential existence of *ycf1* and *rps15* due to contraction and expansion of inverted repeats.

The sizes of the genomes ranged from 163,770 bp (*S. bogneri*) to 169,980 bp (*L. spinose*). The SSC region ranged from 13,967 bp (*O. aquaticum*) to 20,497 bp (*S. bogneri*); LSC ranged from 87,269 bp (*O. aquaticum*) to 91,357 bp (*Z. zamiifolia*); the size of each IR region ranged from 26,702 bp (*S. bogneri*) to 32,053 bp (*L. spinosa*) (Table 2). The chloroplast genomes of the four species were found to be highly conserved in terms of gene organization, gene content, and intron content. This highly conserved structure was also confirmed using circular maps of the genomes (Figure 2) and colinear block analyses of Mauve (Figure 3). All species exhibited 113 unique genes, including 79 protein-coding, 30 tRNA, and 4 rRNA genes. We recorded 17 duplicated genes in the IRs of *S. bogneri* and *Z. zamiifolia* and 18 duplicated genes in *O. aquaticum* and *L. spinosa*. Among duplicated genes, 6–7 were protein-coding, 7 tRNA, and 4 rRNA genes. Hence, the total number of genes ranged from 130 to 131 (Table 2). In total, 17–18 intron-containing genes were observed, including 6 tRNA and 12 protein-coding genes. Among the intron-containing genes, 2 tRNA and 3 protein-coding genes were located in IRs. The size of introns showed some variation among species, whereas exons showed high similarity (Table S2). We detected loss of intron in the *clp*P gene. The *inf*A gene was found to be a pseudogene in all species. The guanine-cytosine (GC) content of the complete chloroplast genomes and of all regions showed high similarities among species, whereas fluctuation in GC content was observed within the different regions of the same chloroplast genome. The GC content of coding regions, rRNAs, and tRNAs also showed high similarities among species (Table 2).

### 3.2. Contraction and Expansion of Inverted Repeats

**Figure 4:**
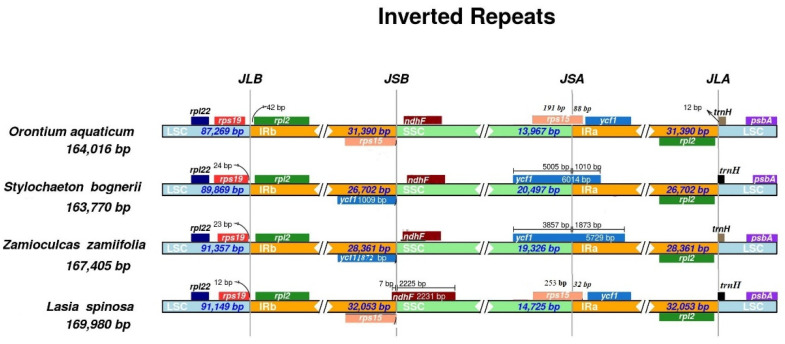
Comparison of quadripartite junction sites among chloroplast genomes of four assembled species. Genes present on top of track transcribe on the negative strand, whereas genes present below the track transcribe on the positive strand. The T scale bar shows integration of genes between two adjacent regions. The junctions of genomes are represented as follows: JLB: IRb/LSC, JSB: IRb/SSC, JSA: SSC/IRa, and JLA: IRa/LSC.

The chloroplast genomes showed overall similarities at the junctions of JLB (LSC/IRb) and JLA (IRa/LSC) across all four species. At JLA in *O. aquaticum*, *trnH-GUG* was found to be completely in the LSC region, 12 bp away from the junction, whereas other species showed integration of *trn*H-GUG into the IRa region from 6 bp to 11 bp. Notable differences were found at the junctions of JSB and JSA among the species. The chloroplast genomes *O. aquaticum* and *L. spinosa* were found to be similar at these two junctions, and IR expansion led to duplication of the complete *ycf*1 gene and the origin of pseudogenes of *rps*15 at JSB. The chloroplast genomes of *S. bogneri* and *Z. zamiifolia* showed less expansion of IRs, which led to the inclusion of only a pseudogene of *ycf*1 at JSB. The integration of *ndh*F into IRb was only recorded in *L. spinosa*. The complete details are presented in Figure 4. 

### 3.3. Analyses of Codon Usage, Amino Acid Frequency and RNA Editing 

The codon usage analyses revealed high encoding efficacy for those codons that end with A/T as opposed to codons that end with C/G. We recorded a relative synonymous codon usage (RSCU) value ≥ 1 for most codons that end with A/T, whereas RSCU < 1 was recorded for codons that end with C/G (Table S3). The ATG codon is the most common start codon. However, we also observed ACG (in *rpl*2) and GTG (in *rps*19) as start codons. The amino acid frequency analyses revealed high encoding of leucine, whereas the rarest encoding was recorded for cysteine (Figure S1). The RNA editing analyses revealed the presence of 62–74 RNA editing sites in 19–21 genes (Table S4). The RNA editing sites were found in the same genes with a few exceptions: RNA editing sites were detected in *psa*B genes of only *Z. zamiifolia*, whereas the RNA editing site was not found in *rpl*20 of *S. bogneri* or *rps*8 of *L. spinosa*. Most of the RNA editing sites were recorded in *ndh*A, *ndh*B, *ndh*D, *rpo*A, *rpo*B, *rpo*C1, and *rpo*C2 (Table S4). ACG was found as a start codon in gene *rpl2* and the RNA editing analyses confirmed conversion of the ACG codon to ATG. Most RNA editing sites were found to be related to conversion of serine to leucine. Moreover, almost all editing sites led to accumulation of hydrophobic amino acids in the polypeptide chain of proteins (Table S4).

### 3.4. Repeats Analyses

**Figure 5:**
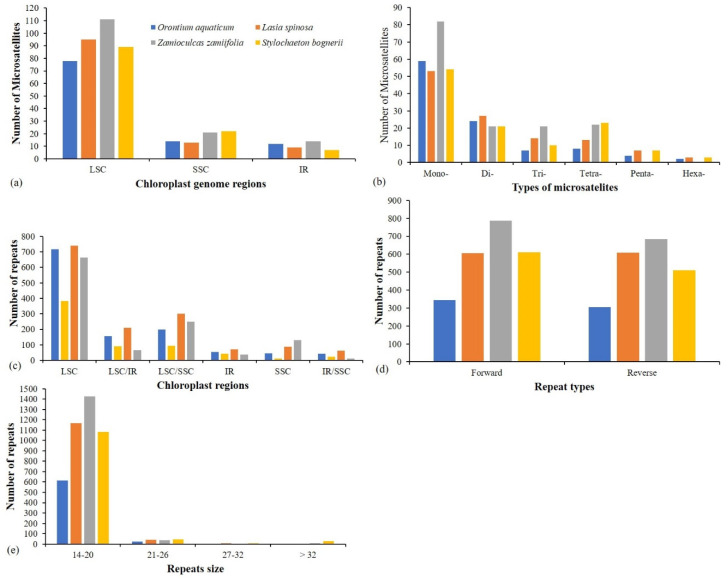
Comparison of repeats among chloroplast genomes of four species. (**a**) Microsatellites distribution in regions of chloroplast genomes. (**b**) Numbers of different types of microsatellites. (**c**) Distribution of oligonucleotide repeats in regions of chloroplast genomes. (**d**) Types of oligonucleotide repeats. (**e**) Number of repeats based on size. LSC: large single copy, SSC: small single copy, IR: inverted repeats; LSC/SSC, LSC/IR, and SSC/IR represent those repeat pairs in which one copy exists in one region and another copy in another region. 14–20, 21–26, 27–32, and >32 showed a range of repeat sizes.

The analyses of microsatellites revealed 104–146 repeats in the genomes. Most of the repeats existed in LSC, followed by SSC and then IR (Figure 5a). Mononucleotide repeats were most abundant in all species, especially in *Z. zamiifolia*. Dinucleotide repeats were in greater abundance in *O. aquaticum* and *L. spinosa*, whereas *S. bogneri* and *Z. zamiifolia* showed an abundance of mononucleotide repeats and tetranucleotide repeats. *Lasia spinosa*, *Z. zamiifolia,* and *O. aquaticum* showed similarities in numbers of trinucleotide and tetranucleotide repeats in their respective genomes, but *S. bogneri* showed few trinucleotide repeats relative to tetranucleotide repeats (Figure 5b). Pentanucleotide and hexanucleotide repeats were in lower abundance than the other types of repeats and were completely lacking in *Z. zamiifolia* (Figure 5b). Most repeats of all six microsatellite types were of the A/T motif rather than the G/C motif (Table S5). The analyses of oligonucleotide repeats revealed the existence of a higher number of forward and reverse repeats in all four species. We recorded most repeats in the LSC region as compared with the SSC and IR regions. We also found some shared repeats among the three regions of the chloroplast genomes (Figure 5c). The number of repeats ranged from 647 (*O. aquaticum*) to 1471 (*Z. zamiifolia*). We recorded high similarities in the numbers of forward and reverse repeats in *O. aquaticum* and *L. spinosa*, whereas in *S. bogneri* and *Z. zamiifolia* there was a higher abundance of forward repeats (Figure 5d). Most repeats ranged in length from 14 to 20 bp (Figure 5e), whereas the largest repeats varied from 39 bp (*L. spinosa*) to 75 bp (*S. bogneri*). Details about the position and number of repeats are provided in Table S6. The oligonucleotide repeats were not linked to subfamily-level classification or to unisexual or bisexual clade-based divisions.

### 3.5. Analyses of Substitution Types

We recorded a greater number of *Ts* substitutions than *Tv* substitutions. The ratios of *Ts/Tv* were 2.3, 2.03, and 2.15 in the genomes of *L. spinosa*, *S. bogneri*, and *Z. zamiifolia*, respectively. The majority of *Ts* substitutions were promoted by A/G rather than by C/T, whereas the majority of *Tv* substitutions were found to be related to A/C and G/T rather than to A/T and C/G (Table 3). For *K_s_* and *K_a_*, we found a higher average of *K_s_* than *K_a_*. Hence, on average, we recorded very low *K_a_/K_s_* for all genes, which shows that purifying selection has acted on these genes. The average values recorded for the different groups of genes were as follows: photosystem I group (*K_s_* = 0.1677, *K_a_* = 0.0125, and *K_a_/K_s_* = 0.1211), photosystem II group (*K_s_* = 0.1208, *K_a_* = 0.0085, and *K_a_/K_s_* = 0.0671), cytochrome group (*K_s_* = 0.1757, *K_a_* = 0.0298, and *K_a_/K_s_* = 0.2012), ATP synthase group (*K_s_* = 0.1466, *K_a_* = 0.0188, and *K_a_/K_s_* = 0.1337), ribosomal small subunit group (*K_s_* = 0.1519, *K_a_* = 0.0589, and K_a_/K_s_ = 0.3584), ribosomal large subunit group (*K_s_* = 0.1480, *K_a_* = 0.0614, and *K_a_/K_s_* = 0.4640), NADPH dehydrogenase group (*K_s_* = 0.1771, *K_a_* = 0.0780, and *K_a_/K_s_* = 0.3855), and RNA polymerase group (*K_s_* = 0.1813, *K_a_* = 0.3228, and *K_a_/K_s_* = 0.1800) (Table S7). Some genes, including *ndh*K, *pet*L, *rpl*16, *ndh*F, *ndh*H, and *rps*15, showed neutral selection (*K_a_/K_s_* = 1) in all species. Interestingly, we found evidence for positive selection in three genes (*ycf*2, *clp*P, and *rpl*36) in only *S. bogneri* (Table S7). 

### 3.6. Phylogenetic Inference of Araceae

**Figure 6:**
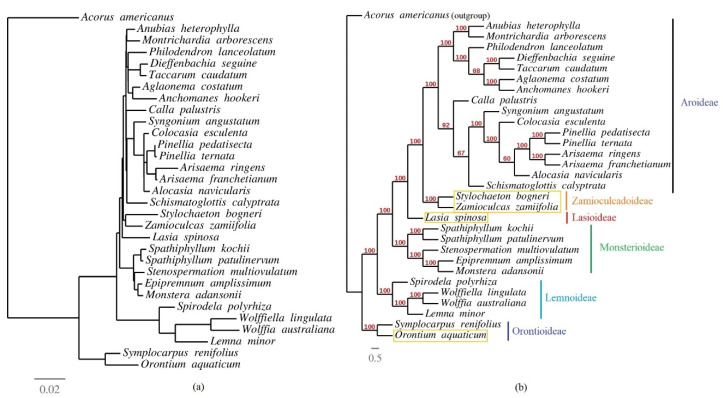
Maximum likelihood tree based on multiple alignment of 30 species of Araceae. (**a**) Phylogenetic tree; (**b**) cladogram. The bootstrapping support values are similar for the phylogenetic tree and the cladogram and are shown only on the cladogram for easy visualization. The four species reported in the current study are highlighted with yellow boxes.

A maximum likelihood phylogenetic tree was reconstructed using RAxML-HPC BlackBox v.8.2.12 in CIPRES using 30 species of Araceae and one outgroup (*A. americanus*). The alignment had 93,821 nucleotides in which 13,486 sites were parsimony informative, 13,060 were singletons, and the remaining sites (67,275) were shared in all species. The resulting phylogeny shows the monophyly of Lasioideae, Zamioculcadoideae, and Orontioideae, with Zamioculcadoideae forming a clade with *Stylochaeton* (Figure 6).

## 4. Discussion

In the current study, we report de novo assembled and fully annotated chloroplast genomes of four species from three subfamilies of Araceae. Comparative chloroplast genomics revealed high similarities in gene content across all species. However, the sizes of these genomes varied due to the variable length of intergenic spacer (IGS) regions and IR contraction and expansion. Substitution analyses revealed *Ts* > *Tv* and *K_s_* > *K_a_*. The phylogenetic analysis confirmed the monophyly of Lasioideae, Orontioideae, and Zamioculcadoideae. 

The chloroplast genomes are either conserved [10,11,12,19] or highly polymorphic in terms of gene content and gene organization [6,14,63,64]. The chloroplast genomes of the four species showed a highly conserved structure of gene content, intron content, and gene organization. Similar gene contents were also reported in other subfamilies of Araceae [8,11,19,36,42]. These observations are in accordance with the growing body of literature showing highly malleable junction sites within an otherwise highly conserved chloroplast genome structure in Araceae regardless of phylogenetic position, similar to other angiosperms [3,12,20,21]. However, loss of some important protein-coding and tRNA genes has been reported in the genus *Amorphophallus* (Aroideae, Araceae), which might be specific to this genus. The *inf*A gene encodes translation initiation factor I, but we found this gene to be non-functional in all species. This gene is also reported to be non-functional or absent in the chloroplast genomes of other angiosperms, including species of Araceae [10,11,12,19,36,65]. Hence, it is suggested that either this gene is transferred to the nuclear genome as an active functional gene or a functional copy of this important gene already exists in the nuclear genome [39,66]. We observed duplication of *ycf*1 genes or origination of pseudogenes of *ycf*1 and *rps*15 due to IR contraction and expansion. The duplication of *ycf*1 and/or *rps*15 is also reported in species of the subfamily Lemnoideae [36] and two species (*Anchomanes hookeri* Schott. and *Zantedeschia aethiopica* Spreng.) of Aroideae [11].

Araceae is an ancient plant family and belongs to the early Cretaceous period [35]. The Araceae stem lineage is dated to 135 Ma and the crown group to 121.7 Ma [35]. Despite this ancient nature, the chloroplast genomes of its species are conserved and do not show gene rearrangement. However, later diverging families from Araceae, such as the dicot family Fabaceae (74–79 Ma) [67] and monocot family Orchidaceae (~80 Ma) [68], show significant genome rearrangement due to inversions and translocations [1,6,14,69]. Moreover, loss of IR regions and loss of several genes, such as *acc*D, *clp*P, *psa*I, *rpl*33, *rps*16, and *ycf*4, have been reported in Fabaceae [6,70], whereas loss of several *ndh* genes has been described in Orchidaceae [64]. These mutational events are also linked to phylogenetic relationships. So far, chloroplast genomes of up to 35 species are reported from seven subfamilies of Araceae [8,11,19,39,41,42,71]. These species are highly diverse in morphology and ecology [43] and in evolutionary periods, ranging from the early Cretaceous to the Miocene period [35]. However, rearrangements of genes have been described in only *Zantedeschia aethiopica*, a highly cultivated plant [11,43], whereas gene loss is reported in only some species of the genus *Amorphophallus* [18]. The highly conserved chloroplast genomes of Araceae in terms of gene content and arrangement is of special interest and could provide further insight into the mechanisms involved in the process. Repeats are considered mutation-causing agents [39,48,72] and are also found to be associated with the extent of inversions by recombination [73,74]. The current study and previous reports [8,11,19] show the existence of a high number of repeats in the chloroplast genomes of Araceae, but these repeats do not produce rearrangement. The four classes of nuclear-encoded genes—chloroplast mutator (CHM/MSH1), organellar single-stranded DNA-binding proteins, RecA-like homologs, and the Whirlies—are identified to escape mutations and genome rearrangement by suppressing recombination between repeated DNA sequences [75]. The high-level genome rearrangements in *Geraniaceae* are suggested to be due to mutations of these genes [63]. In our study, the highly diverse species in terms of morphology and ecology and with diverse evolutionary times, from the Cretaceous periods to the Miocene period, revealed a conserved genome and did not support the rearrangement of genes due to inversions, showing the existence of these genes in a highly functional form. However, further study of these genes in diverse species of Araceae can broaden insights into the mechanisms by which these species avoid rearrangement.

The IRs provide stability to chloroplast genomes, preventing breaking of the genomes during various stress conditions [76]. However, the contraction and expansion of IRs led to decrease/increase in cp genome size and conversion of single-copy genes to duplicate-copy genes, and vice versa [6,8,12]. Similar phenomena exist in chloroplast genomes of aroids. Here, we observed duplication of *ycf*1 in *O. aquaticum* and *L. spinosa*. The duplication of *ycf*1 and *rps*15 is also reported in species of subfamilies Lemnoideae [36]. Recently, an increase in the size of LSC regions and IRs has been reported in chloroplast genomes of *Pothos scandens* L. [8]. Previously, double-strand break and illegitimate recombination were considered causes of IR expansion and contraction in Mimosoideae [77], which may also be the case for IR contraction and expansion in Araceae, but further research into the mechanisms underlying IR boundary shifts is needed to test this theory.

The contraction and expansion of the IR among some angiosperms was suggested to have phylogenetic signal [6,10,20,78]. However, together with previously published aroid plastomes, genomes reported in the current study reveal that IR contraction and expansion might be a species-specific event, as opposed to synapomorphies of subfamilies. The duplication of *ycf*1 and origination of the *rps*15 pseudogene in *O. aquaticum* are not observed in species of *Symplocarpus* Salisb. ex Nutt. [42], and both genes are found in the SSC region. The complete duplication of *ycf*1 and *rps*15 is observed in Lemnoideae species [36]; in *S. bogneri* and *Z. zamiifolia*, duplication of partial *ycf*1 is observed, whereas *rps*15 exists as a single copy completely in the SSC region. Moreover, complete duplication of *ycf*1 and partial duplication of *rps*15 were observed in *L. spinosa*, similar to *O. aquaticum*. Although the four analyzed species in our study suggest the occurrence of similar IR contraction and expansion based on unisexual and bisexual clades, previous reports preclude this conclusion, as duplication of *rps*15 and/or *ycf*1 has been observed in several unisexual species [11], and the single-copy existence of *rps*15 and *ycf*1 has also been noted in bisexual species [19,41,42]. These data suggest that IR contraction and expansion are highly flexible over evolutionary time, and that similar IR boundary architecture across lineages can be the result of homoplasy. In other angiosperms, differential IR contraction and expansion have also been seen in species of the same genus such as *Aquilaria* Lam. [24,39,79,80]. 

We observed *K_a_/K_s_* < 1 due to higher *K_s_* than *K_a_* for most of the protein-coding genes. These results are consistent with previous studies of angiosperm chloroplast genomes, including the family Araceae, as purifying selection pressure mostly acts on the genes of chloroplast genomes [10,12,19,65]. However, a higher *K_a_/K_s_* was also reported in some species of Araceae in which most of the genes were under positive selection [42]. We found three genes under positive selection in the chloroplast genome of *S. bogneri*, including *ycf*2, *clp*P, and *rpl*36. The signatures of positive selection in chloroplast genes in these species might be due to the different types of stresses that they face in their respective ecological niches. The same genes were found to be under positive selection in various other species as well [12,17,65,81,82]. 

In conclusion, the ancient plant family Araceae has conserved chloroplast genome structures in terms of gene content and gene arrangement compared with the families that diverged up to 50 million years later from Araceae, i.e., Orchidaceae and Fabaceae, which show significant gene rearrangements due to various inversion events. The high number of repeats is not associated with genome rearrangements. Hence, some specific mechanisms exist in these species that keep the chloroplast genome stable. The accurate and highly active function of CHM/MSH1, organellar single-stranded DNA-binding proteins, RecA-like homologs, and the Whirlies might be one of the reasons. The IR contraction and expansion led to an increase in length and duplication of genes or conversion of duplicated genes to a single copy by allowing movement of genes from one region to another. However, inversion of genes appears to be avoided since this can break gene clusters under a specific operon, leading to pseudogenization of genes. The IR contraction and expansion appear to be homoplasious among these taxa, precluding the use of IR architecture in phylogenetic analyses. The in-depth study of the family Araceae can provide insights into the mechanisms that keep chloroplast genomes conserved in angiosperms.

## Figures and Tables

**Table 1:** Quality and quantity of whole genome short reads and coverage depth analyses of de novo assembled genomes.

Species	Data in GB	Whole Genome Reads (millions)	Phred Score	Chloroplast Reads (millions)	Average Coverage	Maximum Coverage	NCBI Accession
*Orontium aquaticum*	5.43	20.86	37.2	0.20	124.8	1347	MT226773
*Lasia spinosa*	8.35	32.00	37.6	1.74	1021	1929	MT226772
*Zamioculcas zamiifolia*	11.3	43.29	35.69	1.29	774.4	1293	MT226775
*Stylochaeton bogneri*	3.31	12.71	37.39	0.15	92.7	749	MT226774

**Table 2:** Genomic features of de novo assembled chloroplast genomes.

Characteristic		*O. aquaticum*	*L. spinosa*	*Z. zamiifolia*	*S. bogneri*
Size (base pair; bp)		164,016	169,980	167,405	163,770
LSC length (bp)		87,269	91,150	91,357	89,869
SSC length (bp)		13,967	18,551	19,326	20,497
IR length (bp)		31,390	32,053	28,361	26,702
Number of genes*		131 (113)	131 (113)	130 (113)	130 (113)
Protein-coding genes*		85 (79)	85 (79)	84 (79)	84 (79)
tRNA genes*		37 (30)	37 (30)	37 (30)	37 (30)
rRNA genes*		8 (4)	8 (4)	8 (4)	8 (4)
Duplicate genes		18	18	17	17
GC content	Total (%)	37.3	36.1	35.9	35.7
	LSC (%)	35.7	33.9	34.2	34.0
	SSC (%)	31.9	31.0	30.4	29.5
	IR (%)	40.6	41.8	39.7	40.5
	CDS (%)	37.7	37.8	37.5	37.9
	rRNA (%)	55.2	54.6	55.0	55.0
	tRNA (%)	53.1	52.9	53.2	53.1
	All gene %	39.2	39.3	39.0	39.2

* The number of total genes is presented without parentheses, whereas the number of unique genes appears in parentheses.

**Table 3:** Transition and transversion substitutions in protein-coding genes.

Substitution Type	*Lasia spinosa*	*Zamioculcas zamiifolia*	*Stylochaeton bogneri*
A/C	479	467	562
C/T	1376	1269	1459
A/G	1432	1316	1543
A/T	275	254	316
C/G	160	156	195
G/T	305	322	407
*Ts/Tv*	2.3	2.15	2.03

## Supplementary Materials

The following are available online at https://www.mdpi.com/2223-7747/9/6/737/s1: Figure S1: Amino Acids Frequency, Table S1: GenBank accession number of the species used in phylogenetic inference; Table S2: The lengths of introns and exons in the intron containing genes of de novo assembled species; Table S3: Relative synonymous codon usage analyses in the chloroplast genome of newly assembled species; Table S4: RNA Editing Sites Analysis; Table S5: Types of microsatellites motifs and number of various types of repeats in the chloroplast genomes; Table S6: Oligonucleotide repeats in chloroplast genomes of newly assembled species; and Table S7: Evolution rate of protein coding genes.
