# Supplementary material for: Comparison of Chloroplast Genomes among Species of Unisexual and Bisexual Clades of the Monocot Family Araceae
Source: Plants (Basel). 2020 Jun 11;9(6):737. doi: 10.3390/plants9060737 (PMC7355861; doi:10.3390/plants9060737)
Supplement: Supplementary file 1 [file plants-09-00737-s001.zip › Table S2.docx]

**Table S2. The lengths of introns and exons in the intron containing genes of de novo assembled species**

***Lasia spinosa***

______________________________________________________________________________

Gene Strand Start End Exon I Intron I Exon II Intron II Exon III

______________________________________________________________________________

*trnK-UUU* - 1990 4625 37 2563 36

*rps16* - 5338 6617 42 1043 237

*trnS-CGA* + 10923 11692 31 709 60

*atpF* - 13657 15152 145 950 401

*rpoC1* - 22858 25649 423 740 1629

*ycf3* - 46574 48637 126 751 226 806 155

*trnL-UAA*  + 52052 52665 35 529 50

*trnV-UAC*  - 57196 57866 39 579 37

*clpP* - 76300 78379 71 836 292 638 243

*petB* + 81336 82783 6 800 642

*petD* + 83066 84292 8 744 475

*rpl16* - 87923 89496 9 1166 399

*rpl2* - 91204 92685 391 663 431

*ndhB* - 102157 104358 771 675 756

*trnE-GAU* + 109987 110998 32 940 40

*trnA-UGC*  + 111063 111934 38 799 35

*ndhA* - 134199 136379 553 1089 539

*trnA-UGC*  - 149196 150067 37 799 36

*trnE-GAU* - 150132 151143 32 940 40

*ndhB* + 156772 158973 771 675 756

*rpl2* + 168442 169926 391 663 431

***Orontium aquaticum***

_____________________________________________________________________________

Gene Strand Start End ExonI IntronI ExonII IntronII ExonIII

_____________________________________________________________________________

*trnK-UUU* - 1642 4198 38 2520 36

*rps16* - 4851 6403 42 1324 228

*trnS-CGA* + 9907 10648 31 710 60

*atpF* - 12660 14028 145 814 410

*rpoC1* - 21848 24656 423 757 1629

*ycf3* - 44842 46857 126 760 226 749 155

*trnL-UAA*  + 50107 50786 35 595 50

*trnV-UAC*  - 54298 54973 33 584 59

*clpP* - 72743 74828 71 809 292 671 243

*petB* + 77782 79231 6 802 642

*petD* + 79434 80662 8 744 475

*rpl16* - 85607 84102 9 1098 399

*rpl2* - 87312 88802 391 669 431

*ndhB* - 97835 100067 777 700 756

*trnE-GAU* + 105697 106720 32 952 40

*trnA-UGC*  + 106785 107656 37 799 36

*ndhA* - 128910 131149 553 1148 539

*trnA-UGC*  - 143630 144501 37 799 36

*trnE-GAU* - 144566 145589 32 952 40

*ndhB* + 151219 153451 777 700 756

*rpl2* + 162484 163974 391 669 431

***Stylochaeton* *bogneri***

___________________________________________________________________________

Gene Strand Start End Exon I IntronI ExonII IntronII ExonIII

_____________________________________________________________________________

*trnK-UUU* - 1750 4388 37 2567 35

*rps16* - 5206 6507 42 1065 195

*trnS-CGA* + 11330 12126 31 706 60

*atpF* - 14079 15447 145 823 401

*rpoC1* - 23322 26104 423 731 1629

*ycf3* - 46272 48299 126 755 226 766 155

*trnL-UAA*  + 51856 52450 35 510 50

*trnV-UAC*  - 57084 57748 33 575 57

*clpP* - 76709 77305 71 809 292 671 231

*petB* + 80140 81614 6 802 642

*petD* + 81815 88197 8 744 475

*rpl16* - 86499 84102 9 1098 399

*rpl2* - 89919 91404 391 664 431

*ndhB* - 100740 102947 777 675 756

*trnE-GAU* + 108625 109641 32 945 40

*trnA-UGC*  + 109706 110581 37 803 36

*ndhA* - 127518 129721 556 1109 539

*trnA-UGC*  - 143059 143934 37 803 36

*trnE-GAU* - 143999 145015 32 945 40

*ndhB* + 150693 152900 777 675 756

*rpl2* + 162236 163721 391 664 431

***Zamioculcas zamiifolia***

______________________________________________________________________________

Gene Strand Start End ExonI IntronI ExonII IntronII ExonIII

______________________________________________________________________________

*trnK-UUU* - 1830 4471 38 2568 36

*rps16* - 5258 6572 42 1079 195

*trnS-CGA* + 11667 12461 31 704 60

*atpF* - 14417 15777 145 815 401

*rpoC1* - 23434 26216 423 731 1629

*ycf3* - 46092 48122 126 759 226 765 155

*trnL-UAA*  + 52011 52631 35 536 50

*trnV-UAC*  - 56682 57347 39 592 35

*clpP* - 76327 78418 71 827 292 659 243

*petB* + 81297 82740 6 802 642

*petD* + 82942 84194 8 744 475

*rpl16* - 87775 89712 9 1098 399

*rpl2* - 91413 92898 391 664 431

*ndhB* - 103176 105385 777 677 756

*trnE-GAU* + 110980 111990 32 939 40

*trnA-UGC*  + 112055 112932 37 805 36

*ndhA* - 130541 132791 553 1159 539

*trnA-UGC*  - 145831 146708 37 805 36

*trnE-GAU*  - 146773 147783 32 939 40

*ndhB* + 153378 155587 777 677 756

*rpl2* + 165865 167350 391 664 431
