# Supplementary figures and images for: Comparison of Chloroplast Genomes among Species of Unisexual and Bisexual Clades of the Monocot Family Araceae
Source: Plants (Basel). 2020 Jun 11;9(6):737. doi: 10.3390/plants9060737 (PMC7355861; doi:10.3390/plants9060737)

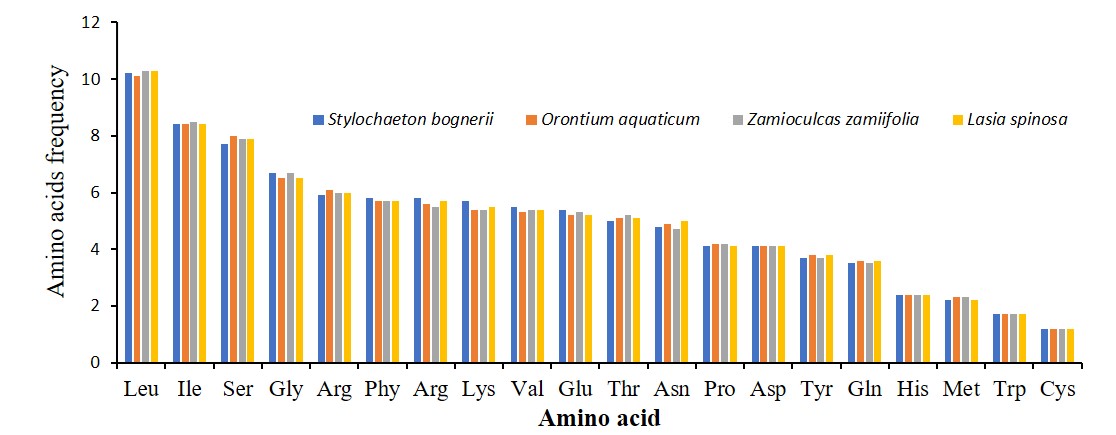

Supplement: Supplementary file 1 [file plants-09-00737-s001.zip › Figure S1.jpg]
